# High-Maintenance-Dose Clopidogrel in Patients Undergoing Percutaneous Coronary Intervention: A Systematic Review and Meta-Analysis

**DOI:** 10.1371/journal.pone.0078549

**Published:** 2013-10-23

**Authors:** Yu Chen, Yachen Zhang, Yong Tang, Xiaohong Huang, Yuquan Xie

**Affiliations:** Division of Cardiology, Xinhua Hospital School of Medicine, Shanghai Jiaotong University, Shanghai, China; Temple University, United States of America

## Abstract

**Background:**

Despite routine use of clopidogrel, adverse cardiovascular events recur among some patients undergoing percutaneous coronary intervention (PCI). To optimize antiplatelet therapies, we performed a meta-analysis to quantify the efficacy of high versus standard-maintenance-dose clopidogrel in these patients.

**Methods:**

Randomized controlled trials (RCTs) comparing high (>75 mg) and standard maintenance doses of clopidogrel in patients undergoing PCI were included. The primary efficacy and safety end-points were major adverse cardiovascular/cerebrovascular events (MACE/MACCE) and major bleeding. The secondary end-points were other ischemic and bleeding adverse effects. The pooled odds ratio (OR) for each outcome was estimated.

**Results:**

14 RCTs with 4424 patients were included. Compared with standard-maintenance-dose clopidogrel, high-maintenance-dose clopidogrel significantly reduced the incidence of MACE/MACCE (OR 0.60; 95% CI 0.43 to 0.83), stent thrombosis (OR 0.56; 95% CI 0.32 to 0.99) and target vessel revascularization (OR 0.38; 95% CI 0.20 to 0.74), without significant decrease of the risk of cardiovascular death (OR 0.92; 95% CI 0.74 to 1.13) and myocardial infarction (OR 0.83; 95% CI 0.51 to 1.33). For safety outcomes, it did not significantly increase the risk of major bleeding (OR 0.73; 95% CI 0.41 to 1.32), minor bleeding (OR 1.29; 95% CI 1.00 to 1.66) and any bleeding (OR 1.14; 95% CI 0.91 to 1.43).

**Conclusion:**

High-maintenance-dose clopidogrel reduces the recurrence of most ischemic events in patients post-PCI without increasing the risk of bleeding complications.

## Introduction

Clopidogrel, a P2Y12 adenosine diphosphate (ADP) receptor antagonist, can inhibit platelet aggregation, which has been demonstrated that it can reduce the risk of recurrent cardiovascular events in patients with acute coronary syndromes (ACS) [1,2]. In the current guidelines it is recommended as an indispensable segment in antithrombotic therapy in patients undergoing percutaneous coronary intervention (PCI) [3,4]. However, high on-treatment platelet reactivity (HTPR) exists under routine dosage of clopidogrel among some patients, which is often called as clopidogrel resistance or nonresponsiveness [5,6]. Studies have revealed that HTPR is associated with the recurrence of major adverse cardiovascular events (MACE) post-PCI [7,8]. To overcome HTPR and optimize the antiplatelet therapies in patients post-PCI, several treatment strategies have been tested recent years, such as choice of new generation ADP-receptor antagonists (prasugrel, ticagrelor) and increase of clopidogrel dosage [9,10]. Prasugrel and ticagrelor have been demonstrated that they can significantly reduce the risk of ischemic events compared to standard-dose clopidogrel in patients with ACS [11,12]. And they are suggested as preferred antiplatelet agents by the European Society of Cardiology (ESC) [13]. However, the higher risk of bleeding and greatly increased cost constrains their wide use. To increase the loading or maintenance dose of clopidogrel is an alternative choice. A number of studies demonstrated that high-loading-dose clopidogrel (600mg) reduced the risk of cardiovascular death (CV death) or myocardial infarction (MI) in 30-day duration post-PCI [14-16]. And Siller-Matula et al performed a meta-analysis to find that high-loading-dose clopidogrel reduced the rate of MACE without increase in major bleeding compared to the standard-loading-dose clopidogrel in patients undergoing PCI during one month follow-up [17]. Besides, some studies have investigated the feasibility and benefit of high-maintenance-dose clopidogrel in patients undergoing PCI, but their verdicts were inconsistent. Therefore, in this study we performed a systematic review and meta-analysis of all available data to quantify the clinical evidences on the efficacy and safety of high-maintenance-dose clopidogrel in patients undergoing PCI.

## Methods

This review was written according to the Preferred Reporting Items for Systematic Reviews and Meta-Analyses statement (Checklist S1) [18] and Cochrane Collaboration guidelines [19].

### Search strategy

PUBMED (from 1966 to August 2013), EMBASE (from 1974 to August 2013), and the Cochrane Central Register of Controlled Trials (CENTRAL) (Issue 7, 2013) were searched for pertinent RCTs with the following search strategies. Relevant keywords relating to clopidogrel (“clopidogrel” or “plavix” or “iscover” [Title/Abstract]) were used in combination with words relating to clopidogrel dosage (“high” or “higher” or “double” or “150 mg” [All Fields]) and words relating to PCI (“coronary intervention” or “PCI” or “stent*” or “angioplasty” [Title/Abstract]) . No language restrictions were applied. Furthermore, an extensive manual search was performed. We referred relevant original articles, reviews, editorials, and letters on this topic. Useful data not reported in the original papers were acquired by communicating with the authors. In addition, we searched websites for recent trials (www.clinicaltrial.gov, www.cardiosource.com, www.controlled-trials.com). 

### Inclusion and exclusion criteria

Studies were included if they met the following criteria: (1) Randomized controlled trials comparing high-maintenance-dose clopidogrel (>75mg) versus standard-maintenance-dose clopidogrel (75mg), with equivalent loading dose of clopidogrel, standard-dose aspirin and follow-up ≥30 days; (2) patients with coronary atherosclerosis heart disease (CAD) and undergoing PCI. The exclusion criteria were: (1) ongoing studies, (2) duplicate reports (3), unpublished studies (with data unavailable from the principal investigators) (4), studies with incomplete follow-up.

### Outcome measures

The primary efficacy end-point was the incidence of MACE or major adverse cardiovascular and cerebrovascular events (MACCE), which was defined as composite events of CV death, MI, target vessel revascularization (TVR), stent thrombosis (ST) and stroke. The secondary efficacy end-points were the rate of CV death, MI, TVR, or ST. Major bleeding was chosen as primary safety end-point. Minor bleeding and any bleeding were considered as secondary safety end-points. 

### Data collection and quality assessment

All data were independently extracted with a standardized data extraction form by two investigators (YC, and YT). Results were compared, and disagreements were resolved by discussion with a third investigator (YZ). For each RCT, the following data were abstracted: leading author’s name, year of publication, location, age, gender, patterns of stent type, patterns of coronary atherosclerosis heart disease (CAD), main past medical history, concomitant medication, number of patients, number of patients with clinical events, intervention strategy, duration of follow-up, and efficacy and safety outcomes of the treatment. The qualities of included studies were assessed by the risk of bias in accordance with the Cochrane Collaboration methods [19]. In detail we evaluated information regarding sequence generation, incomplete outcome data addressing, allocation concealment, blinding, selective reporting and other biases. No formal scoring system was used. Reviewers were not blinded to journal, author, or institution of publication.

### Statistical analysis

Analyses were performed with Review Manager 5.1 (The Cochrane Collaboration, Oxford, United Kingdom), Stata 12.0 (StataCorp, College Station, TX) and Comprehensive Meta Analysis 2.2 (Biostat, Englewood, NJ, USA). The κ statistic was used to assess agreement between reviewers for study selection. The measure of treatment effect for each study was the odds ratio (OR) with 95% confidence interval (CI). The overall treatment effect was estimated by the pooled OR with 95% CI using a fixed-effect model (Mantel Haenszel) or a random-effect model (DerSimonian-Laird). Heterogeneity was evaluated by means of I^2^ test, which quantifies the percentage of the variability that is due to heterogeneity rather than chance. Values above 25%, 50%, and 75% were assigned to low, moderate, and high degree of heterogeneity [20,21]. Sensitivity analysis was used to take into account the influence of study quality. Publication bias was assessed by Egger's test, fail-safe number (Nfs0.05) and a funnel plot of effect size against standard error [22,23]. After publication bias was found, the Duval and Tweedie's trim and fill method was used to impute “hypothetical” missing studies and to calculate adjusted versus observed ORs. A two-tailed *p* value < 0.05 was considered statistically significant for each test.

## Results

### Eligible studies

**Figure 1:**
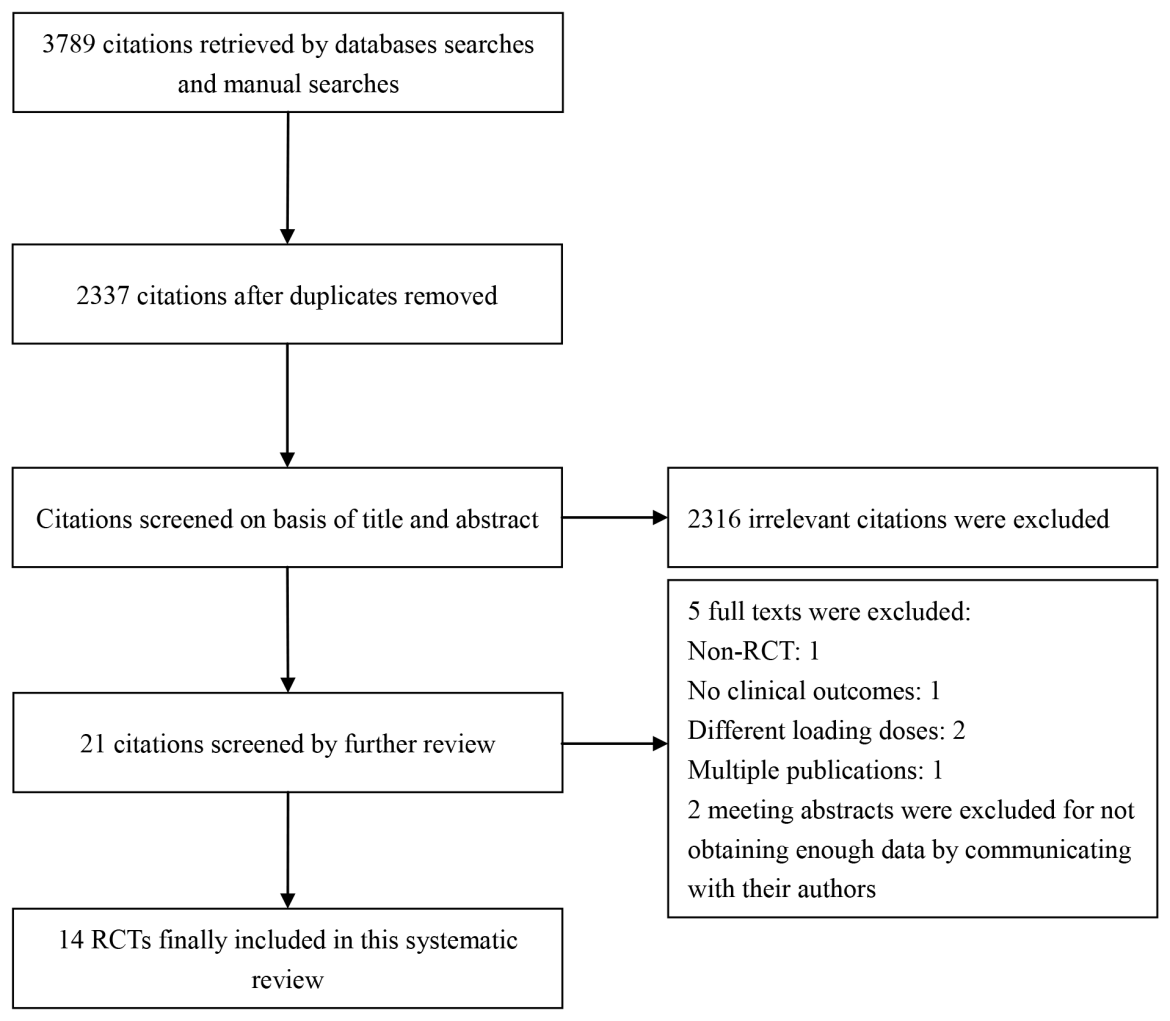
Flow chart of study selection.

**Figure 2:**
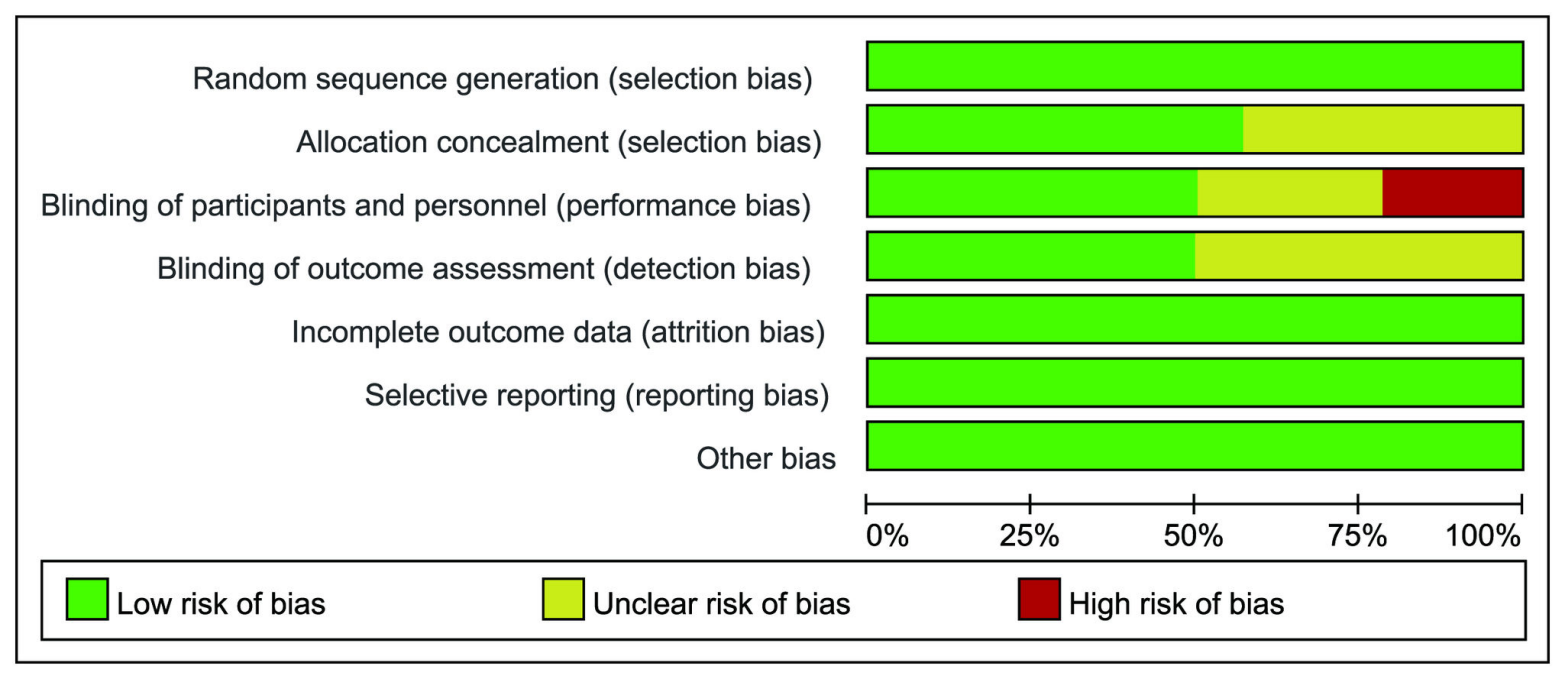
Risk of bias graph.

Study selection process is presented in Figure 1. Total 3789 citations were retrieved by database and manual searches. 1452 duplicates and 2316 irrelevant citations were excluded by title and abstract evaluation. 5 studies were excluded for not fulfilling to our inclusion criteria by detailed full-text screening and 2 meeting abstracts for not obtaining enough data by communicating with their authors. At the end 14 RCTs with a total of 4424 patients were included in this systematic review [24-37]. The interobserver agreement for study selection was good, with a κ value of 0.80. The detailed characteristics of the 14 RCTs are given in Table 1, Table 2. The methodological qualities of included studies were assessed by the risk of bias (Figure 2). Among them six trials were found to have a low risk of bias [24,25,29,31,33,36], five with an unclear risk of bias [28,30,32,34,35] and three with a high risk of bias [26,27,37]. 

**Table 1:** Main characteristics of the included RCTs.

RCTs	Location	Patients (High/standard)	Intervention	Included end-points	Follow-up
Angiolillo 2008[25]	USA and Spain	20/20	600 mg LD immediately after PCI, 150mg vs. 75mg MD for 30 days, then 75mg MD for both for another 30 days	MACE, bleeding complications	60 days
Aradi 2012[33]	Hungary	36/38	600 mg LD before PCI, 150 vs. 75mg MD for 1mo, then 75mg MD for both until 12 mo	MACE, CV death, MI, TVR, TIMI major/minor bleeding	12 mo
ARMYDA-150mg 2011[36]	Italy	25/25	600 mg LD before PCI, 75mg MD for 30 days, then 150mg vs. 75mg MD for another 30 days	MACCE, death, MI, TVR, ST, stroke, bleeding complications	2 mo
DOUBLE 2010[28]	Italy	24/24	300mg LD before PCI, then 150mg vs. 75mg MD for 30 days	ST, bleeding complications	30 days
EFFICIENT 2011[34]	Turkey	47/47	After PCI 150mg vs. 75mg MD for 1 mo, then 75mg for 6 mo	MACCE, CV death, MI, ACS, ST, TVR, stroke, TIMI major/minor bleeding	6 mo
GRAVITAS 2011[29]	USA and Canada	1109/1105	After PCI 150mg MD vs. 75mg MD for 6mo	MACE, CV death, MI, ST, bleeding complications	6 mo
Gremmel 2011[31]	Austria	21/23	300/600mg LD before PCI, then 150mg vs. 75mg MD for 3 mo	ST, in-stent restenosis, bleeding complications	3 mo
Han 2009[27]	China	403/410	600 mg LD before PCI, 150mg vs. 75mg MD for 30 days	MACE, CV death, MI, ST, TVR, TIMI major/minor bleeding	30 days
Ren LH 2012[32]	China	46/55	300mg LD before PCI, 150mg vs. 75 mg MD for 30 days, then 75mg MD for both until 6 mo	MACE, CV death, MI, TVR, TIMI major/minor bleeding	6 mo
Roghani 2011[35]	Iran	205/195	600 mg LD before PCI, 150mg vs. 75mg MD for 30 days	MACE, CV death, MI, ST, bleeding complications	1 mo
Tousek 2011[37]	Czech	30/30	12-24 hours after PCI with 600mg LD, maintenance doses were increased in a stepwise manner according to PRU(>240) vs. 75mg for 30 days	MACCE, death, MI, stroke, TIMI major/minor bleeding	6 mo
VASP-02 2008[26]	France	58/62	300mg/600mg before PCI, 150mg vs. 75mg MD for 28 days	MACCE, CV death, MI, TVR, stroke, major/minor bleeding	28 days
von Beckerath 2007[24]	Germany	31/29	600 mg LD before PCI, 150mg vs. 75mg MD for 30 days	MI, TVR, TIMI major/minor bleeding	30 days
Wang 2011[30]	China	150/156	300 mg LD before PCI, maintenance doses were increased in a stepwise manner according to VASP-PRI (up to 375 mg) vs. 75mg for 12mo	MACE, CV death, MI, ACS, ST, TVR, TIMI major/minor bleeding	12 mo

Abbreviations: RCT: randomized controlled trial; LD: loading dose; MD: maintenance dose; MI: myocardial infarction; ACS: acute coronary syndrome; ST: stent thrombosis; MACE: major adverse cardiac events; MACCE, major adverse cardiac and cerebrovascular events; CV: cardiovascular; TVR: target vessel revascularization; TIMI: thrombolysis in myocardial infarction criteria; PRU: P2Y12 reaction units; VASP: vasodilator-stimulated phosphoprotein; PRI: platelet reactivity index.

**Table 2:** Main characteristics of the included RCTs (continued).

RCTs	Age (mean)	Male	CAD pattern	HTPR	DM	LVEF	Smoking	GPI	PPI	Statin	Stent type
Angiolillo 2008[25]	63	68%	SCAD100%	NA	33%	NA	30%	0	NA	100%	BMS/DES
Aradi 2012[33]	62	53%	SCAD100%	100%	43%	NA	36%	0	27%	72%	BMS/DES
ARMYDA-150mg 2011[36]	63	84%	Stable angina: 36%, UA/NSTEMI: 64%	NA	40%	56%	0	0	NA	100%	BMS/DES
DOUBLE 2010[28]	63	90%	STEMI 100%	NA	17%	NA	67%	100%	46%	65%	BMS
EFFICIENT 2011[34]	58	77%	SCAD100%	100%	29%	57%	66%	2%	28%	62%	BMS
GRAVITAS 2011[29]	64	65%	STEMI: <1%, UA/NSTEMI: 39%,SCAD: 60%	100%	45%	29%	14%	0	30%	77%	DES
Gremmel 2011[31]	68	68%	SCAD100%	100%	45%	NA	41%	0	55%	95%	BMS/DES
Han 2009[27]	64	74%	UA/USTEMI 25%,STEMI:75%	NA	31%	56%	39%	0	NA	51%	DES
Ren LH 2012[32]	68	NA	SCAD100%	NA	72%	53%	24%	0	NA	NA	BMS/DES
Roghani 2011[35]	60	66%	SCAD100%	NA	19%	NA	19%	0	NA	NA	BMS/DES
Tousek 2011[37]	66	75%	Stable angina: 23.3%, UA/NSTEMI: 33.3%, STEMI43.3%	100%	30%	47%	60%	0	NA	NA	BMS/DES
VASP-02 2008[26]	65	82%	SCAD100%	NA	24%	NA	16%	4%	27%	78%	BMS/DES
von Beckerath 2007[24]	64	92%	SCAD100%	NA	28%	NA	8%	0	NA	98%	BMS/DES
Wang 2011[30]	67	70%	Stable angina: 80%, UA/NSTEMI: 20%	100%	43%	55%	39%	0	NA	100%	DES

Abbreviations: RCT: randomized controlled trial; CAD: coronary atherosclerosis heart disease; HTPR: high on-treatment platelet reactivity; DM: diabetes mellitus; LVEF: left ventricular ejection fraction; GPI: glycoprotein IIb/IIIa inhibitor; PPI: Proton Pump Inhibitor; SCAD: stable coronary atherosclerosis heart disease; UA: unstable angina; STEMI: ST-segment elevation myocardial infarction; NSTEMI: non-ST segment elevation myocardial infarction; BMS: bare-metal stent; DES: drug-eluting stent; NA: not applicable

### Clinical end-points

**Figure 3:**
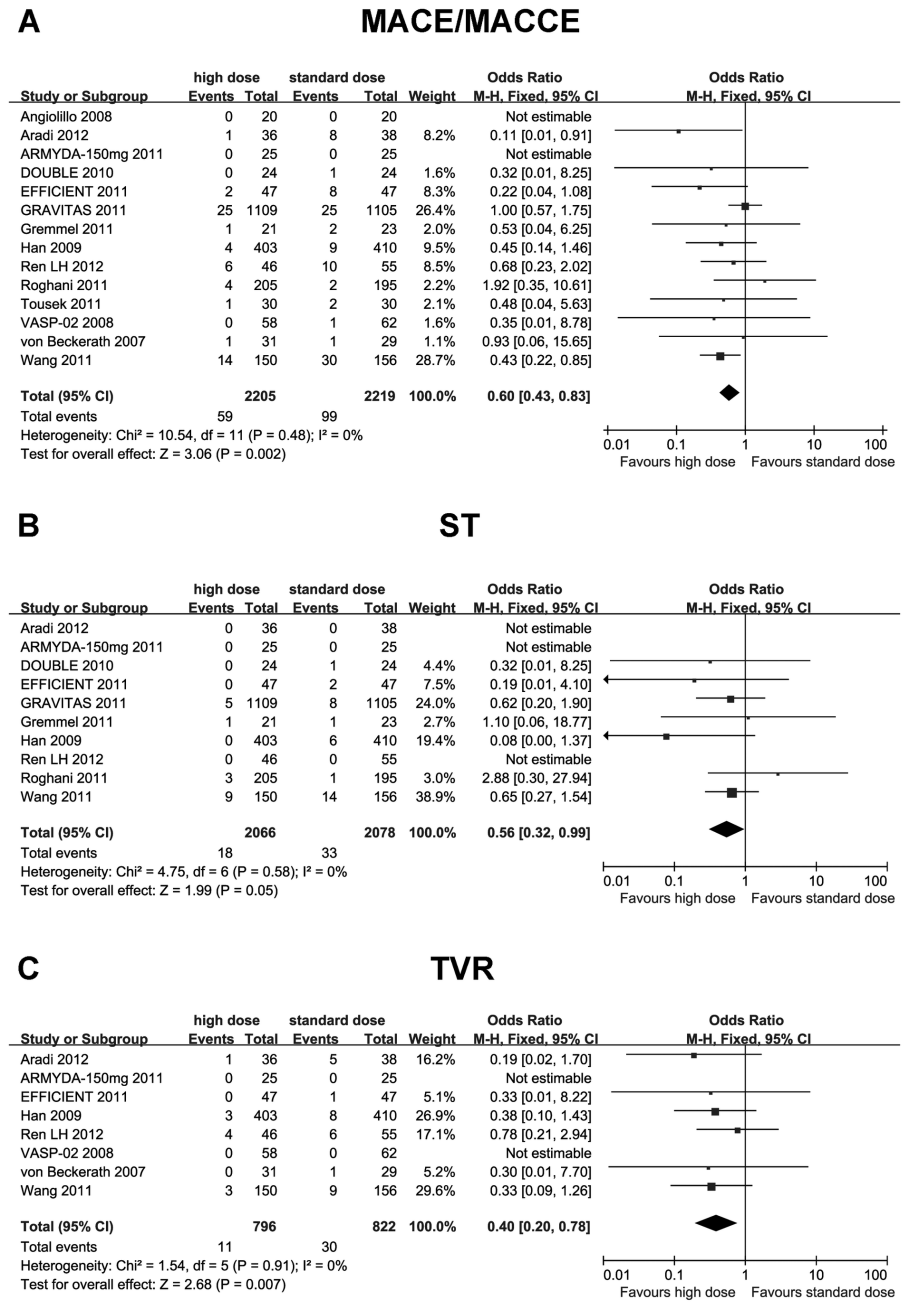
Comparisons of high versus standard maintenance-dose clopidogrel on MACE/MACCE, ST and TVR. A: MACE/MACCE; B: ST; C: TVR. MACE: major adverse cardiac events; MACCE, major adverse cardiac and cerebrovascular events; ST: stent thrombosis; TVR: target vessel revascularization.

**Figure 4:**
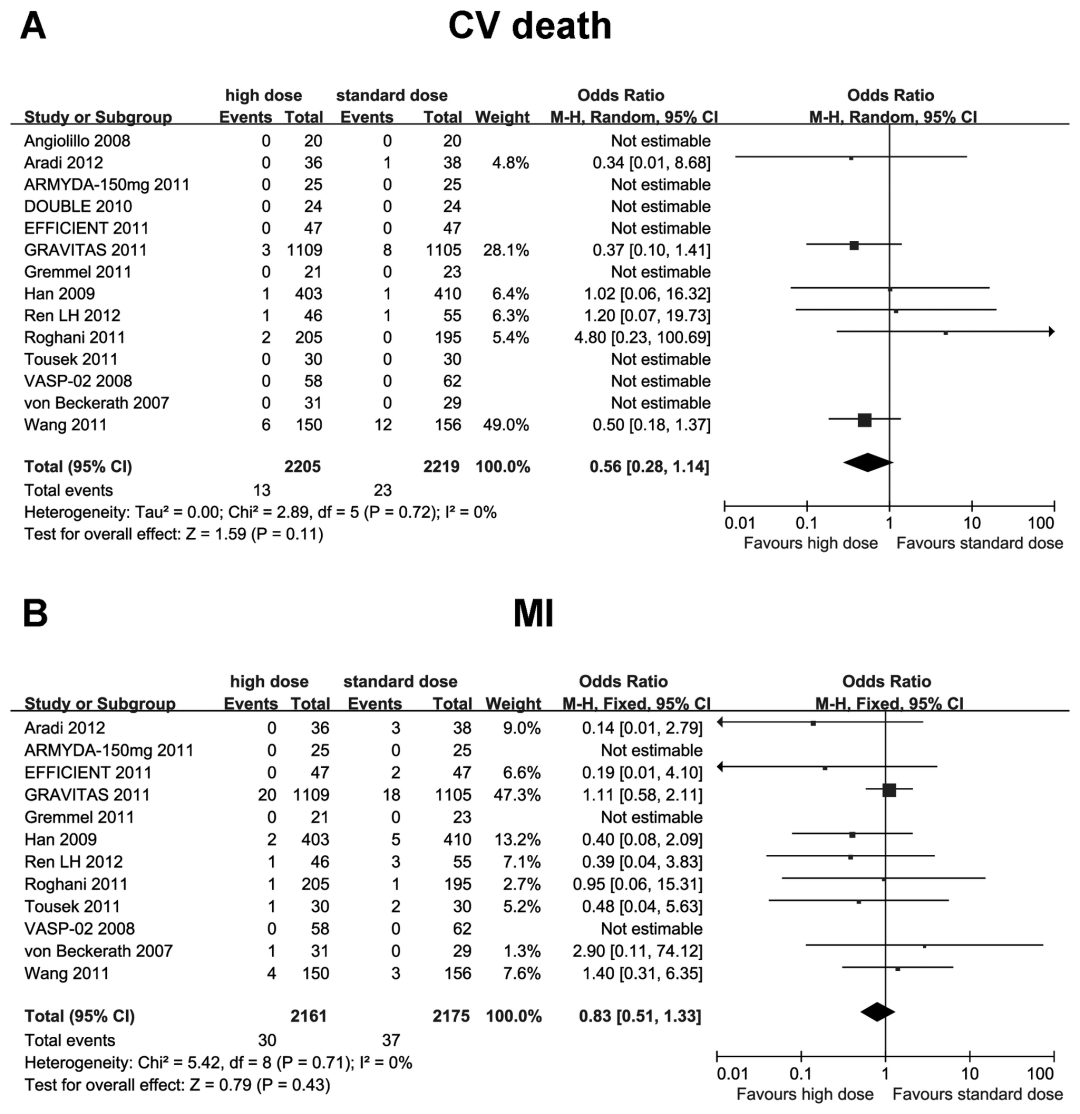
Comparisons of high versus standard maintenance-dose clopidogrel on CV death and MI. A: CV death; B: MI. CV death: cardiovascular death; MI: myocardial infarction.

In comparison with standard-dose clopidogrel, high-maintenance-dose clopidogrel significantly reduced the risk of MACE/MACCE (OR 0.60; 95% CI 0.43 to 0.83; I^2^ =0%; *p* = 0.002), ST (OR 0.56; 95% CI 0.32 to 0.99; I^2^ =0%; *p*=0.049), and TVR (OR 0.38; 95% CI 0.20 to 0.74; I^2^ =0%; *p*=0.004) (Figure 3). The risk of CV death and MI reduced too, but did not achieve statistical significance (CV death: OR 0.92; 95% CI 0.74 to 1.13; I^2^ =0%; *p*=0.40; MI: OR 0.83; 95% CI 0.51 to 1.33; I^2^ =0%; *p*=0.43) (Figure 4). 

**Figure 5:**
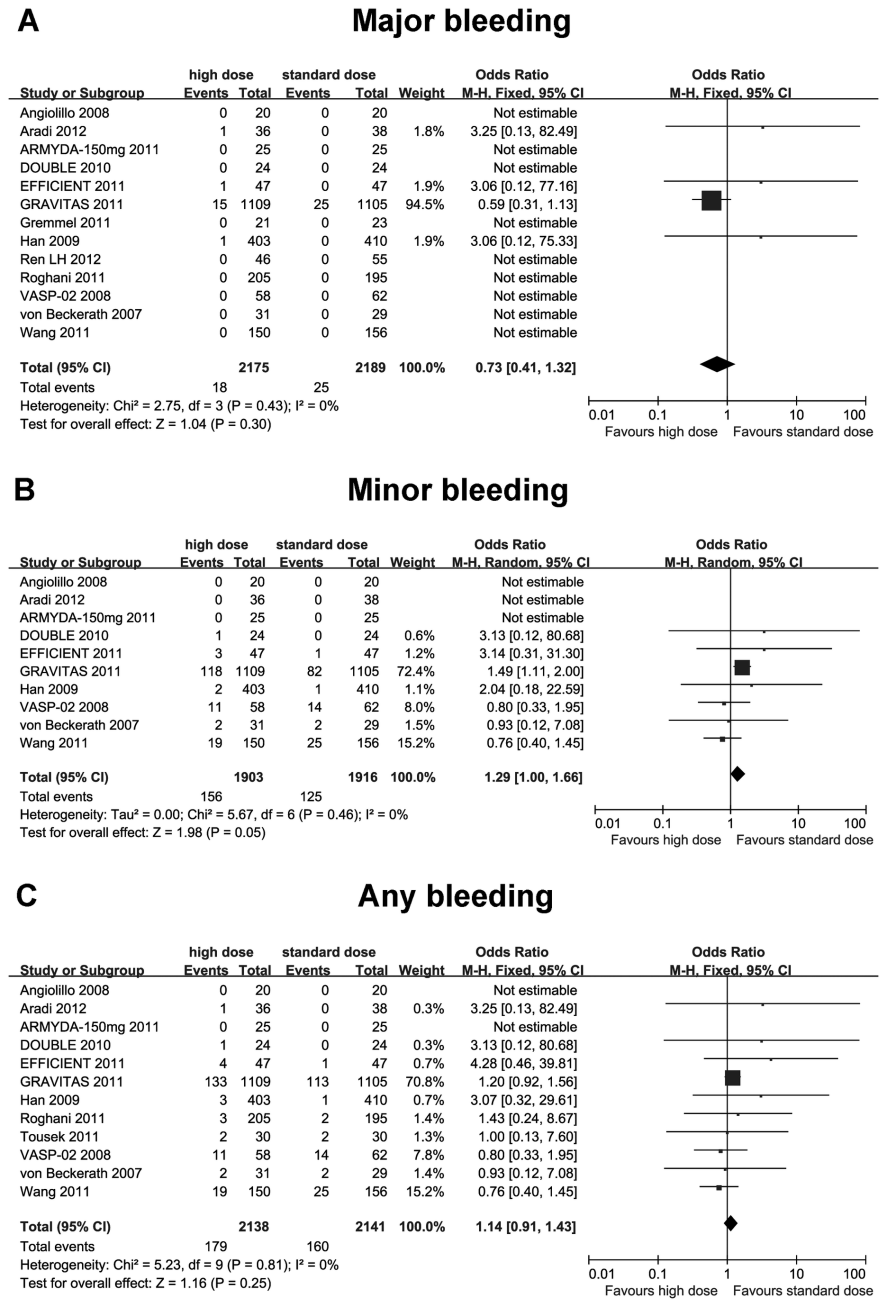
Comparisons of high versus standard maintenance-dose clopidogrel on bleeding complications. A: major bleeding; B: minor bleeding; C: any bleeding.

For safety end-points, high-maintenance-dose clopidogrel did not significantly increase the incidence of major bleeding (OR 0.73; 95% CI 0.41 to 1.32; I^2^ =0%; *p*=0.30), minor bleeding (OR 1.29; 95% CI 1.00 to 1.66; I^2^ =0%; *p*=0.05) and any bleeding (OR 1.14; 95% CI 0.91 to 1.43; I^2^ =0%; *p*=0.25) (Figure 5).

### Subgroup analyses

Subgroup analyses were performed in terms of follow-up duration, maintenance dose, loading dose, stent type, CAD type and HTPR among the included end-points (Table 3). All the subgroups experienced reduced MACE/MACCE, although several subgroups such as 1month, 600mg LD and ACS did not reach statistical significance. The major bleeding, minor bleeding or any bleeding did not increase in all the subgroups except for a mild increase of minor bleeding in fixed dose subgroup.

**Table 3:** Subgroup and sensitivity analyses.

	MACE/MACCE	*p* *	Major bleeding	*p* *	Minor bleeding	*p* *	Any bleeding	*p* *
***Treatment duration***		0.79		0.37		0.63		0.90
1month	0.66 (0.29, 1.48)		3.06 (0.12, 75.33)		0.96 (0.46, 2.04)		1.10 (0.56, 2.15)	
2-12month	0.58 (0.41,0.84)		0.69 (0.38, 1.26)		1.21 (0.69, 2.13)		1.15 (0.90, 1.46)	
***Maintenance dose***		0.27				0.08		0.19
fixed-dose (150mg) vs. 75mg	0.67 (0.45, 0.98)		0.73 (0.41, 1.32)		1.42 (1.08, 1.86)		1.21 (0.95, 1.55)	
stepwise increasing dose (≥150mg) vs. 75mg	0.44 (0.23, 0.84)		No		0.76 (0.40, 1.45)		0.78 (0.42, 1.44)	
***Loading dose***		0.97				0.58		0.45
300mg	0.48 (0.27, 0.85)		No		0.80 (0.43, 1.51)		0.81 (0.43, 1.52)	
600mg	0.49 (0.24, 1.02)		3.15 (0.32, 30.70)		1.29 (0.27, 6.08)		1.53 (0.60, 3.89)	
***Stent type***		0.17		0.35		0.33		0.19
BMS	0.23 (0.06, 0.98)		3.06 (0.12, 77.16)		3.13 (0.48, 20.49)		3.89 (0.62, 24.38)	
DES	0.66 (0.45, 0.99)		0.64 (0.34, 1.20)		1.19 (0.71, 2.01)		1.13 (0.89, 1.45)	
***CAD type***		0.87		0.99		0.39		0.33
ACS	0.43 (0.14, 1.30)		3.06 (0.12, 75.33)		2.37 (0.34, 16.40)		3.09 (0.48, 19.82)	
SCAD	0.48 (0.25, 0.90)		3.16 (0.32, 30.98)		0.95 (0.44, 2.05)		1.16 (0.60, 2.25)	
***HTPR***		0.71		0.37		0.63		0.90
Yes	0.49 (0.26, 0.94)		0.69 (0.38, 1.26)		1.21 (0.69, 2.13)		1.14 (0.90, 1.46)	
NA	0.66 (0.35, 1.27)		3.06 (0.12, 75.33)		0.96 (0.46, 2.04)		1.10 (0.56, 2.15)	
***Trial quality***		0.53		0.37		0.43		0.69
Low or unclear risk of bias	0.62 (0.44, 0.88)		0.69 (0.38, 1.26)		1.28 (0.92, 1.80)		1.16 (0.91, 1.47)	
High risk of bias	0.44 (0.16, 1.21)		3.06 (0.12, 75.33)		0.90 (0.39, 2.06)		0.99 (0.47, 2.09)	

Abbreviations: MACE: major adverse cardiac events; MACCE, major adverse cardiac and cerebrovascular events; BMS: bare-metal stent; DES: drug-eluting stent; CAD: coronary atherosclerosis heart disease; HTPR: high on-treatment platelet reactivity; SCAD: stable coronary atherosclerosis heart disease; ACS: acute coronary syndrome; NA: not applicable; p* value was calculated for subgroup comparison.

### Heterogeneity assessment and sensitivity analyses

All the I^2^ values of the included end-points were 0%, which indicated that there was no heterogeneity for each included end-point. Further, sensitivity analyses were performed by excluding RCTs with a high risk of bias [26,27,37], showing no significant change in the overall treatment effect of all end-points (Table 3). Besides, in order to detect the small-study effect, we compared the results between the fixed-effect model and random-effect model in accordance with the Cochrane Collaboration methods [19], and did not find that the latter was significantly superior to the former*.*

### Publication bias

**Figure 6:**
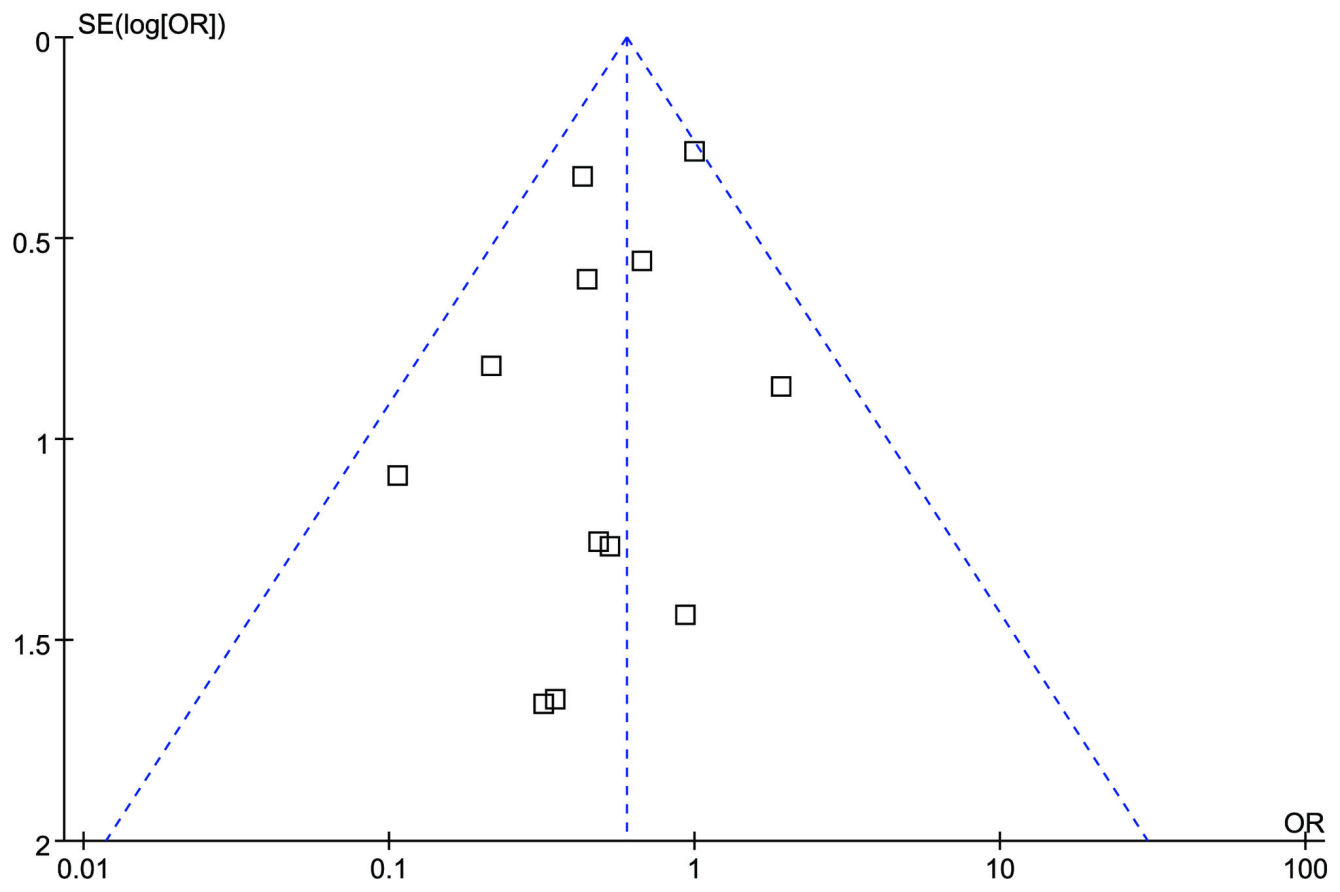
Funnel plots of MACE/MACCE for the comparison of high versus standard maintenance-dose clopidogrel. MACE: major adverse cardiac events; MACCE, major adverse cardiac and cerebrovascular events.

The publication bias of included trials was assessed by funnel plots, Nfs0.05 and Egger's test of MACE/MACCE. The funnel plot of MACE/MACCE was mild asymmetric, but the Duval and Tweedie's trim and fill method did not detect any “hypothetical” missing studies (Figure 6). The Nfs0.05 of MACE/MACCE was 48, which implied another 48 negative studies would be needed to invalid the present effect size. In view of only 14 eligible RCTs were retrieved, the possibility to miss so many negative RCTs was very small. Egger's test of MACE/MACCE did not show skewed distribution (*p*=0.25), which indicated the publication bias was not significant. 

## Discussion

The main findings of this review could be summarized as follows: (1) Compared with standard-dose clopidogrel, high-maintenance-dose clopidogrel significantly reduced the risk of MACE/MACCE, ST and TVR; (2) High-maintenance-dose clopidogrel did not significantly increase the risk of major bleeding, minor bleeding and any bleeding*.*

The antiplatelet activity of clopidogrel was dose-dependent. High-dose clopidogrel can generate more intense inhibition of platelet function. Besides, some other effects of clopidogrel have been found recent years. Ren et al showed that clopidogrel could retard the progression of established lesions by inhibiting inflammation and cell proliferation, and promotion of cell apoptosis [38]. Waksman et al demonstrated that clopidogrel reduced inflammation and neointimal formation in balloon-denuded arteries of hypercholesterolemic rabbits [39]. Heitzer et al found that clopidogrel improved endothelial nitric oxide bioavailability and diminished biomarkers of oxidant stress and inflammation in patients with symptomatic coronary artery disease [40]. The reduced rates of ischemic events, including MACE/MACCE, ST and TVR, may be related to these pleotropic effects of clopidogrel. Besides, the effects of clopidogrel are influenced by some clinical factors. Our subgroup analyses suggested that long-term use, stepwise doses, or HTPRs could get more benefits from high-maintenance-dose clopidogrel. By contrast, its effect is independent of stent types. Both bare-mental stent and drug-eluting stent can benefit significantly from high-maintenance-dose clopidogrel. 

In despite of intensified antiplatelet activity, high-maintenance-dose clopidogrel did not increase the risk of major bleeding, verifying the long-term safety in patients with PCI. Besides, all the subgroups did not experience increased major bleeding, minor bleeding or any bleeding except for a mild increase of minor bleeding in the fixed dose subgroup. Compared to the fixed dose subgroup, the stepwise dose subgroup did not experience increased bleeding events, suggesting stepwise-dose manner is safer and superior for patients with PCI. This may be because stepwise doses according to VASP-PRI/PRU can avoid excessive inhibition of platelet function. 

Responsiveness to clopidogrel varies widely among individuals [41-43]. Studies revealed that HTPR or clopidogrel resistance exists in 10-30% of patients using clopidogrel [14,44]. How to vanquish HTPR remains to be a challenge to cardiovascular researchers and physicians. Six RCTs with HTPR patients were included in this systemic review [29-31,33,34,37]. Subgroup analyses demonstrated that HTPRs obtained significant benefit of reduced recurrent ischemic events from high-maintenance-dose therapy and they did not present with more bleeding events. Actually, high-maintenance-dose clopidogrel trends to be utilized in HTPRs. Among them the platelet reactivity to clopidogrel was relatively low and high-dose clopidogrel provided only a modest amount of incremental platelet inhibition [29]. Thus the bleeding risk of HTPRs is lower than that of non-HTPRs. Furthermore, stepwise doses according to VASP-PRI/PRU may be a safer and effective strategy for HTPRs post-PCI. 

Nowadays new generation ADP-receptor antagonists have gained predominant concerns. Large RCTs such as TRITON–TIMI 38 Trial and PLATO Trial demonstrated that prasugrel or ticagrelor was superior to standard clopidogrel therapy in prevention of ischemic events in ACS patients with scheduled PCI [11,12]. As a result, they were recommended as preferred options for the management of acute ST-elevation myocardial infarction (STEMI) in patients with scheduled PCI in ESC guidelines [13]. It seems the era of novel ADP-receptor antagonists has come and clopidogrel will be replaced completely in future. However, growing questions and controversies are emerging. Firstly, the increased rate of bleeding complications accompanied by greater costs caused by the novel ADP-receptor antagonists cannot be neglected. Recently, Roe et al found that compared with clopidogrel, prasugrel did not significantly reduce the frequency of the composite events of CV death, MI, or stroke among patients with unstable angina or MI without ST-segment elevation [45]. Furthermore, despite lack of data of direct comparisons between novel ADP-receptor antagonists and high dose clopidogrel, the indirect comparisons performed by Steiner Sabine et al showed that prasugrel or ticagrelor did not exhibit significant superiority to high-maintenance-dose clopidogrel in reducing the recurrence of ischemic events except for stent thrombosis [46]. In addition, our meta-analysis demonstrates that high-maintenance-dose clopidogrel can significantly reduce the risk of MACE/MACCE without increasing the rate of bleeding complications compared to standard-dose clopidogrel. Thus, even apart from economic factor, clopidogrel should be kept as a fundamental and first-line regimen of antiplatelet therapy for patients undergoing PCI before more powerful clinical evidences preferring novel thienopyridine are obtained.

There are several limitations in our systemic review. Firstly, as well as other meta-analyses, heterogeneity caused by different clinical factors is a common and unavoidable limitation. Fortunately, the heterogeneities of clinical outcomes in this review were not significant, and did not influence our overall conclusion. Secondly, the majority of trials are small-size. These small-size trials may be present with some potential risks like inadequate balance after randomization, which is called “small-study effect” in other words. However, we did not find significant small-study effect by an assessment according to the Cochrane Collaboration. Thirdly, the funnel plot of MACE/MACCE was mild asymmetric. Although Egger's test and Nfs0.05 showed the publication bias was not significant and Tweedie's trim-fill method did not detect any “hypothetical” missing studies, we cannot completely deny the possibility of unpublished studies. 

## Conclusion

Our systematic review and meta-analysis demonstrates that high-maintenance-dose clopidogrel significantly reduces the incidence of MACE/MACCE, ST and TVR in patients undergoing PCI, and did not significantly increase the risk of major bleeding, minor bleeding and any bleeding. These findings enhance the strength of clinical evidences for use of high-maintenance-dose clipodogrel in the long-term treatment post-PCI. However, due to the limitations of included trials, some large-size and prolonged follow-up RCTs are expected in future to provide new and more powerful evidences for this issue.

## Supporting Information

Checklist S1 **PRISMA Checklist.**
(DOC)
